# BMP4 Protects Rat Pulmonary Arterial Smooth Muscle Cells from Apoptosis by PI3K/AKT/Smad1/5/8 Signaling

**DOI:** 10.3390/ijms150813738

**Published:** 2014-08-08

**Authors:** Jian Wu, Zhigang Yu, Dechun Su

**Affiliations:** 1 Department of Cardiology, the First Affiliated Hospital of Dalian Medical University, Dalian 116000, China; E-Mail: jianwu111@126.com; 2 Clinic Technology Center of Dalian Medical University, Dalian 116000, China; E-Mail: zhigyu520@163.com

**Keywords:** pulmonary arterial hypertension, bone morphogenetic protein-4, PI3K/AKT, apoptosis

## Abstract

Bone morphogenetic protein-4 (BMP4), a member of the transforming growth factor β (TGF-β) family of growth factors, is activated and increased under hypoxic conditions, which plays an important role in the progression of pulmonary arterial hypertension (PAH). Previous studies have shown that BMP4 is involved in the regulation of proliferation, differentiation, migration and apoptosis of various cell types. However, the precise mechanisms involved in the regulation of pulmonary artery smooth muscle cells (PASMCs) in PAH are still incompletely understood. It has been reported that AKT is a critical regulator of cell survival and vascular remodeling. Therefore, there may be crosstalk between BMP4 anti-apoptotic processes and PI3K/AKT survival effect in rat PASMCs. To test this hypothesis, we performed confocal, cell viability measurement, mitochondrial potential, real-time polymerase chain reaction (PCR), and Western blot analysis to determine the role of BMP4 on cell survival and apoptosis. We found that hypoxia up-regulated the expression of BMP4. BMP4 promoted cell survival, reduced mitochondrial depolarization, and increased the expression of Bcl-2 and procaspase-3 in PASMCs under serum-deprived condition. These effects were reversed by PI3K/AKT inhibitors (LY294002 and wortmannin). Thus, these findings indicate that BMP4 protects PASMCs from apoptosis at least in part, mediated via the PI3K/AKT pathway.

## 1. Introduction

Vasoconstriction, remodeling of the pulmonary vessel wall, and thrombosis *in situ* are major causes for the elevated pulmonary vascular resistance and increased pulmonary arterial pressure (PAP) found in pulmonary arterial hypertension (PAH) [1,2]. The most important characteristic of pulmonary vascular remodeling in PAH is the change in pulmonary vascular structure associated with medial hypertrophy, which is generally thought to result from by imbalanced proliferation and apoptosis in pulmonary artery smooth muscle cells (PASMCs) [3,4,5,6]. Increased PASMCs proliferation and decreased PASMCs apoptosis can cause thickening of the pulmonary vasculature, which subsequently enhance pulmonary vascular resistance, reduce the inner-lumen diameter of pulmonary arteries, and increase PAP [7]. Bone morphogenetic protein (BMP) belongs to the TGF-β superfamily, playing many diverse functions during proliferation, differentiation, migration, and apoptosis [8]. Bone morphogenetic protein-4 (BMP4) triggers numerous cellular responses through receptors and various intracellular signaling pathways [8,9,10,11]. Bone morphogenetic protein (BMP) family members comprise multifunctional cytokines that are important mediators of pulmonary fibrosis and vascular remodeling [12,13,14]. There is growing evidence that abnormalities of the BMP signaling pathway are linked to the pathogenesis of PAH [4,10,15], and BMP4 has been found to be up-regulated by hypoxia in murine lung tissue and to promote the growth and migration of PASMCs, and thus to promote pulmonary arterial remodeling during the development of chronic hypoxic pulmonary hypertension (CHPH) [12,13,14]. BMPs initiate signaling by binding to a receptor complex containing Type I and Type II receptor kinases and the subsequent activation of Smad-dependent and Smad-independent pathways [16]. It has been demonstrated that BMP4 up-regulated transient receptor potential cation channel (TRPC1), TRPC4, and TRPC6 expression, leading to enhanced store operated calcium entry (SOCE) and elevated basal [Ca^2+^]_i_ in PASMCs [17,18]. However, whether BMP4 is involved in anti-apoptosis of PASMCs and the mechanisms underlying the anti-apoptotic effects of BMP4 are unclear.

It has been demonstrated that the activation of AKT inhibits apoptosis of a variety of cell types *in vitro* [19]. PI3K/AKT has been reported to inhibit cellular apoptosis and to promote cell survival in response to growth factor induction [20]. The survival effects of AKT are involved in inhibition of several pro-apoptotic proteins, including FasL, Bad, and caspase-9 [21,22,23]. The involvement of the PI3K/AKT pathway in the pathogenesis of PAH has been widely studied [24]. Therefore, it is possible that the PI3K/AKT pathway plays a role in vascular smooth cell proliferation and apoptosis, and its abnormality leads to PAH.

In the current study, we demonstrate that BMP4 protects apoptosis of PASMCs through the PI3K/AKT/Smad1/5/8 pathway. Our results show that BMP4 inhibits the apoptosis of PASMCs and attenuates a series of apoptotic events involving mitochondrial dysfunction and caspase-3 activation via PI3K/AKT pathway.

## 2. Results and Discussion

### 2.1. The Expression of Bone Morphogenetic Protein (BMP) and Its Receptors (BMPR1A and BMPR2) in Pulmonary Artery

**Figure 1:**
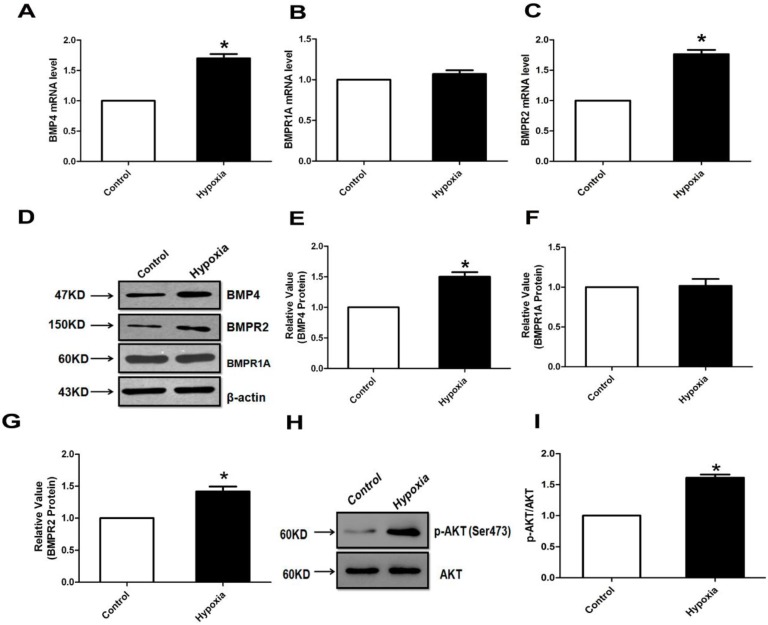
Bone morphogenetic protein 4 (BMP4) and its receptor (BMPR1A and BMPR2) mRNA and protein expression in pulmonary arteries. (**A**–**C**) Quantitative real-time polymerase chain reaction (qPCR) was performed to analyze the BMP4 and its receptor (BMPR1A and BMPR2) expression. Expression levels of target genes were normalized to the β-actin mRNA level using the 2^−ΔΔ*C*t^ method; (**D**–**G**) Western blot analysis of the BMP4 and its receptor (BMPR1A and BMPR2) protein expression; (**H**,**I**) AKT protein expression in rat pulmonary arteries homogenates from normoxic and hypoxic rats. All values are denoted as the mean ± SEM from at least three separate experiments. * *p* < 0.05.

BMP4 and its receptor (BMPR1A and BMPR2) mRNA and protein expression levels in normal and hypoxia pulmonary arteries were evaluated by real-time PCR and Western blotting. BMP4 mRNA and protein expression levels were significantly increased in hypoxia pulmonary arteries compared with controls (Figure 1A,D,E). Intracellular signaling of BMPs occurs via binding to Type I and Type II serine/threonine receptor kinases that then phosphorylate Smad (mainly Smad1, 5 and 8), resulting in the translocation of Smad into the nucleus. Hence, we further studied the expression of its receptors (BMPR1A and BMPR2). We found that BMPR2 mRNA and protein expression levels were significantly up-regulated in hypoxia pulmonary arteries compared with controls (Figure 1C,D,G). However, both mRNA and protein levels of BMPR1A did not change in the normal and hypoxia groups (Figure 1B,D,F). As AKT is a kinase known to promote cell survival and block apoptosis, we further evaluated the regulation of PI3K/AKT signaling during hypoxic PAH. We obtained pulmonary artery samples from rats after 4 weeks of exposure to hypoxia. The expression of p-AKT (Ser473) protein in rat pulmonary arterial homogenates was higher in the hypoxia groups (Figure 1H,I). Meanwhile, BMP4 up-regulated the expression of phosphorylation of AKT1 and AKT2 in a concentration-dependent manner in rat pulmonary arteries and PASMCs (Figure S1A–E).

### 2.2. Hypoxia Induces BMPR Receptors Protein Expression in Human Pulmonary Artery Smooth Muscle Cells (HPASMCs) for Different Time Course

Hypoxia (3% O_2_ concentration) increases HPASMCs BMPR2 protein expression in a time-dependent manner. The maximal increase was observed after 48 h of hypoxia treatment. Protein levels of BMPR1A did not change in HPASMCs in different hypoxic time courses (Figure S2A,B). BMP4 secreted from pulmonary microvascular endothelial cells in response to hypoxia and promoted proliferation and migration of vascular smooth muscle cells, which plays a paracrine role in promoting smooth muscle proliferation and remodeling in hypoxic pulmonary hypertension. To examine whether hypoxia could increase the generation of endogenous BMP4 in human pulmonary artery endothelial cells (HPAECs), the human BMP-4 ELISA (Enzyme-Linked Immunosorbent Assay) kit was carried out for the detection of the amount of BMP4. HPAECs pellets were lyzed and supernatant concentrations were measured by Bradford protein assay. We found that the endogenous BMP4 was increased in a time-dependent manner (Figure S2C).

### 2.3. BMP4 Improved PASMC Viability via the PI3K/AKT Survival Pathway, and Caspase-3 Was Involved in SD-Induced Apoptosis

**Figure 2:**
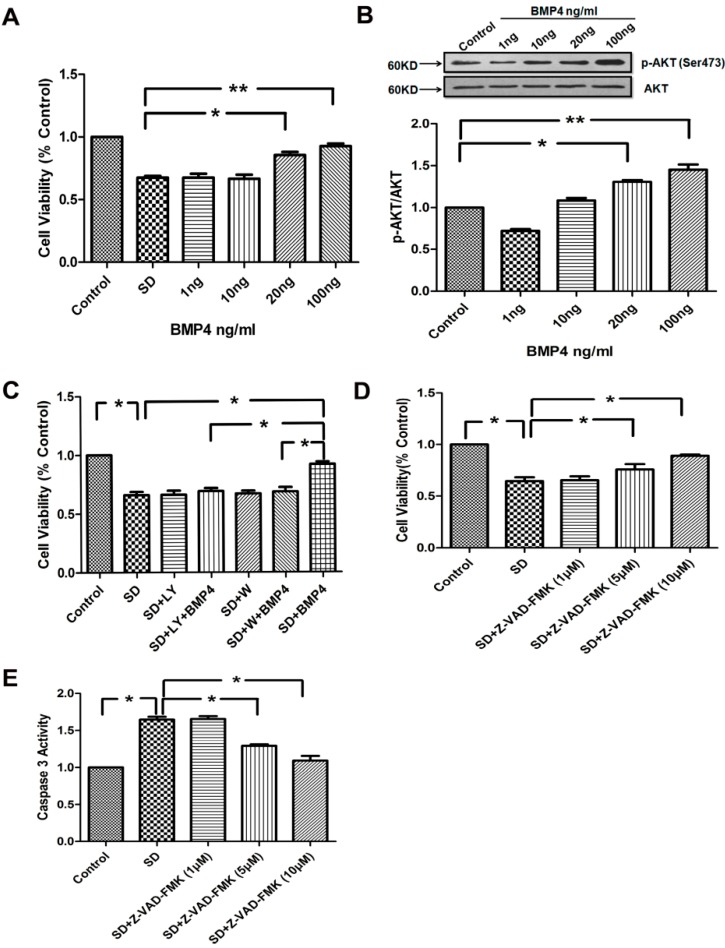
(**A**) Effect of BMP4 on the pulmonary artery smooth muscle cells (PASMCs) survival. Cells were growth-arrested for 24 h and then treated with BMP4 (1–100 ng/mL) subjected to serum withdrawal; (**B**) BMP4 induced activation of AKT in PASMCs. BMP4 up-regulated the expression of phospho-AKT in a concentration-dependent manner; (**C**) BMP4 promoted the survival of PASMCs in AKT-dependent manner. PASMCs were treated as the anticipated groups for 24 h and cell viability was determined by the 3-(4,5-dimethylthiazol-2-yl)-2,5-diphenyltetrazolium bromide (MTT) assay. LY294002 and wortmannin inhibited the protective effect of BMP4 on cell viability in serum deprived conditions; (**D**) Caspase-3 inhibitor Z-VAD–FMK was examined and the caspase-3 pathway contribution to the serum deprivation (SD)-induced apoptosis process was studied using the MTT assay; (**E**) Caspase-3 activity was measured by cleavage of the Ac-DEVD–pNA substrate to pNA. All values are denoted as means ± SEM from three or more independent batches of cells. “SD” means serum deprivation; “LY” means LY294002; “W” means wortmannin. * *p* < 0.05; ** *p* < 0.01.

To investigate the effect of BMP4 on PASMCs viability, we examined the cell viability by measuring colorimetric conversion of 3-(4,5-dimethylthiazol-2-yl)-2,5-diphenyltetrazolium bromide (MTT) to formazan. Serum deprivation caused a marked decrease in PASMCs viability that was partially prevented by a treatment with BMP4 (20 and 100 ng/mL). However, the addition of BMP4 (1 and 10 ng/mL) failed to rescue a significant portion of PASMCs committed to cell suicide (Figure 2A). Hypoxia increases AKT activity in human PASMCs [25]. Phosphoinositide 3-kinase/protein kinase B has been linked to cell survival, transcription factor activation, and multiple signaling pathways. To verify whether similar responses also happened in BMP4 treatment, we treated rat PASMCs with various concentrations of BMP4. As shown in Figure 2B, BMP4 induced AKT phosphorylation in a concentration-dependent manner. The maximal increase was observed with 100 ng/mL BMP4 treatment.

Next, we examined the contribution of the PI3K/AKT pathway to the BMP4 effect on cell viability. We applied BMP4 and the PI3K/AKT inhibitors (LY294002 and wortmannin) to PASMCs. Our results showed that serum deprivation caused a marked decrease in cell viability. The protective effect of BMP4 on cell viability was significantly attenuated by pre-incubation PASMCs with 10 μmol/L LY294002 or 50 nmol/L wortmannin (Figure 2C). All these results indicate that the survival-promoting effect of BMP4 is likely to be mediated by the PI3K/AKT pathway. To understand whether caspase-3 plays a role in the process, we analyzed the cell viability and caspase-3 activity by using the caspase-3 inhibitor Z-VAD–FMK to treat PASMCs. We found that the inhibitor Z-VAD–FMK enhanced the cell viability and suppressed the activity of caspase-3 in a concentration-dependent manner (Figure 2D,E).

### 2.4. The Inhibitory Effects of BMP4 on Caspase-3 Expression and Procaspase-3 Cleavage Were Blocked by PI3K/AKT Inhibitors

**Figure 3:**
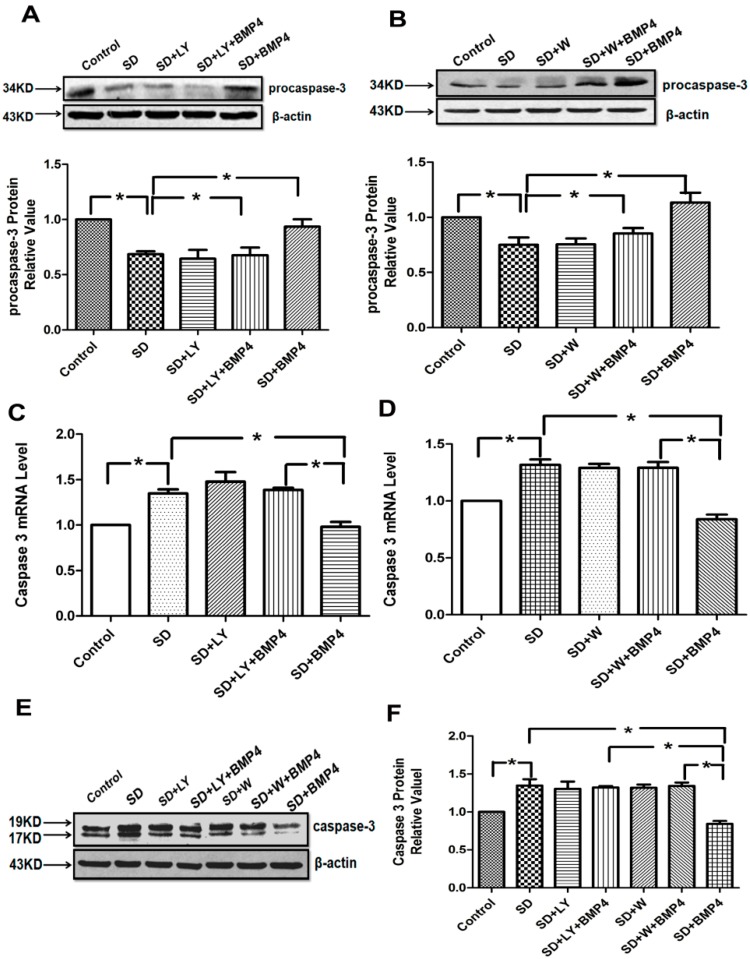
BMP4 suppresses the cleavage of procaspase-3 and the expression of caspase-3 through the PI3K/AKT pathway. (**A**,**B**) BMP4 up-regulated the expression of procaspase-3 in rat PASMCs, and densitometric analysis of the Western blot assays; (**C**,**D**) Quantitative real-time PCR was performed to quantify *caspase-3* expression. Expression levels of target genes were normalized to the *β-actin* mRNA level using the 2^−ΔΔ*C*t^ method; (**E**,**F**) Cleaved caspase-3 expression in rat PASMCs after applying BMP4 and PI3K/AKT inhibitors. All values are denoted as means ± SEM from three or more independent batches of cells. “SD” means serum deprivation; “LY” means LY294002; “W” means wortmannin. * *p* < 0.05.

Caspase-3 is cleaved from procaspase-3 whose expression has been used to indicate caspase-3 activity [26]. We found that serum deprivation (SD) promoted the cleavage of procaspase-3 and induced the expression of caspase-3 as opposed to that in culture medium, and BMP4 significantly inhibited the cleavage of procaspase-3 (Figure 3A,B) and expression of caspase-3 (Figure 3C–F). The effects of BMP4 were reversed by PI3K/AKT inhibitors LY294002 and wortmannin. The results suggest that BMP4 decreases the *caspase-3* mRNA and protein levels by PI3K/AKT signaling.

### 2.5. BMP4 Relieved Mitochondrial Depolarization and Induced Bcl-2 Expression in PASMCs after Serum Deprivation through PI3K/AKT Pathway

**Figure 4:**
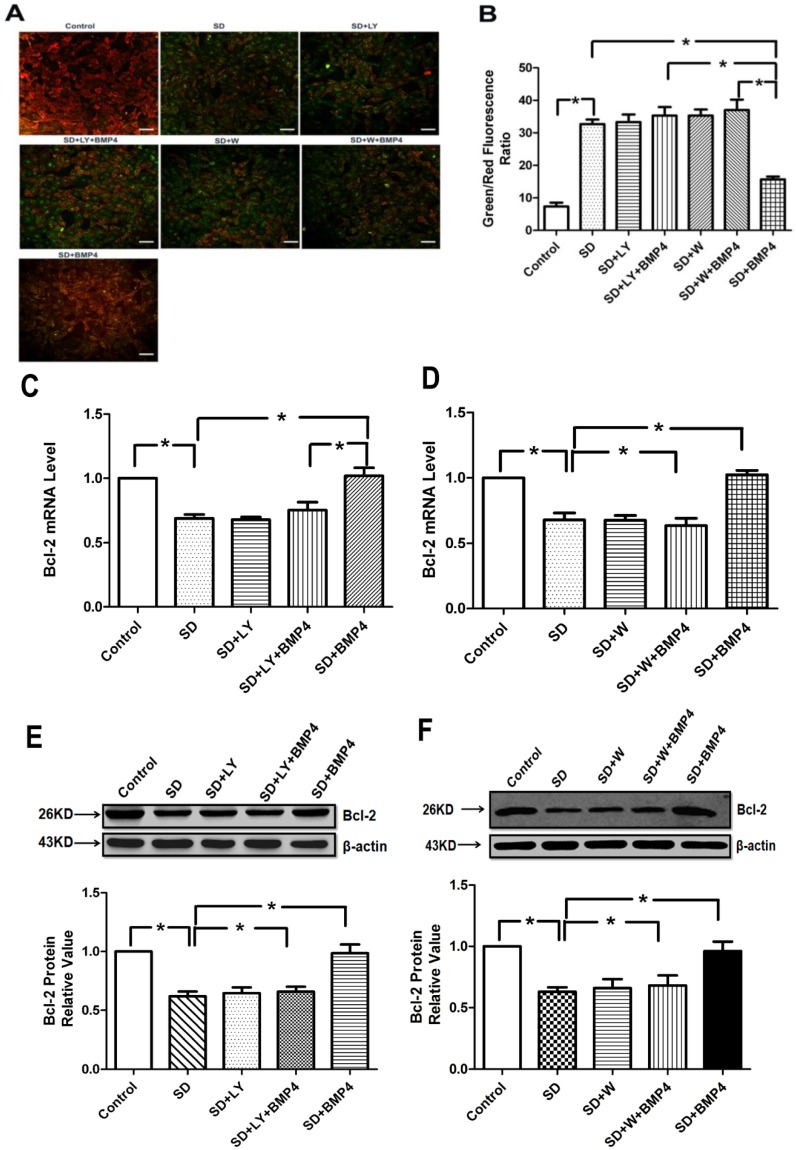
BMP4 relieved mitochondrial potential reduction and induced Bcl-2 expression in PASMCs through the PI3K/AKT pathway. (**A**) The cells were stained with JC-1 probe and imaged by a fluorescentmicroscope. The individual red and green average fluorescence intensities are expressed as the ratio of green to red flintensitie. The increase of fluorescence ratio, which is represented in the bars, correlates with an increase in mitochondrial depolarization. Representative photographs of JC-1 staining in different groups. Scale bar = 10 µm; (**B**) Quantitative analysis of the shift of mitochondrial green fluorescence to red fluorescence among groups; (**C**,**D**) Quantitative real-time PCR was performed to quantify Bcl-2 expression. BMP4 increased the *Bcl-2* mRNA expression induced by serum deprivation through PI3K/AKT pathway. Data were collected from three independent experiments with three to four samples in each; (**E**,**F**) The expression of Bcl-2 increased by BMP4 was partly inhibited by LY294002 and wortmannin (PI3K/AKT inhibitors). All values are denoted as means ± SEM from three or more independent batches of cells. “SD” means serum deprivation; “LY” means LY294002; “W” means wortmannin. * *p* < 0.05.

An important indication of apoptosis is the mitochondrial membrane potential (MOMP), the disruption of which is an early event of apoptosis. 5,5',6,6'-Tetrachloro-1,10,3,30-tetraethylbenzimidazolocarbocyanine iodide (JC-1) was used to assess the changes in MOMP. Normal PASMCs stained with JC-1 emitted mitochondrial orange-red fluorescence with a little green flwith a lit, while in apoptotic PASMCs JC-1 was dispersed to the monomeric form (green fluorescence). The quantitative analysis of JC-1-stained cells showed a significant decrease in the red (high ΔΨm) to green (low ΔΨm) ratio in SD-treated cells when compared with control cells, which were cultured in the presence of 20% fetal bovine serum (FBS). A treatment of SD cells with BMP4 significantly increased the red fluorescence. However, exposure of the LY294002 or wortmannin-treated cells to BMP4 suppressed the effect of BMP4 and did not induce marked changes in ΔΨm in comparison to SD cells (Figure 4A,B). The result showed that BMP4 protected against SD-induced loss of ΔΨm and maintained mitochondrial integrity via PI3K/AKT pathway.

Bcl-2 is one of the important anti-apoptotic Bcl-2 family members participating in the regulation of MOMP [27]. Therefore, we next examined the expression of Bcl-2, which is associated with mitochondrial function. Our data showed that BMP4 up-regulated the Bcl-2 expression both mRNA and protein levels, and that the PI3K/AKT signal transduction pathway participated in this process (Figure 4C–F). These results provide evidence that up-regulated Bcl-2 expression may serve as a critical mechanism involved in BMP4-inhibited apoptosis in PASMCs, and that the PI3K/AKT pathway plays a key role.

### 2.6. BMP4 Activates Smad1/5/8 Phosphorylation by the PI3K/AKT Signaling Pathways in Pulmonary Arterial Smooth Muscle Cells

**Figure 5:**
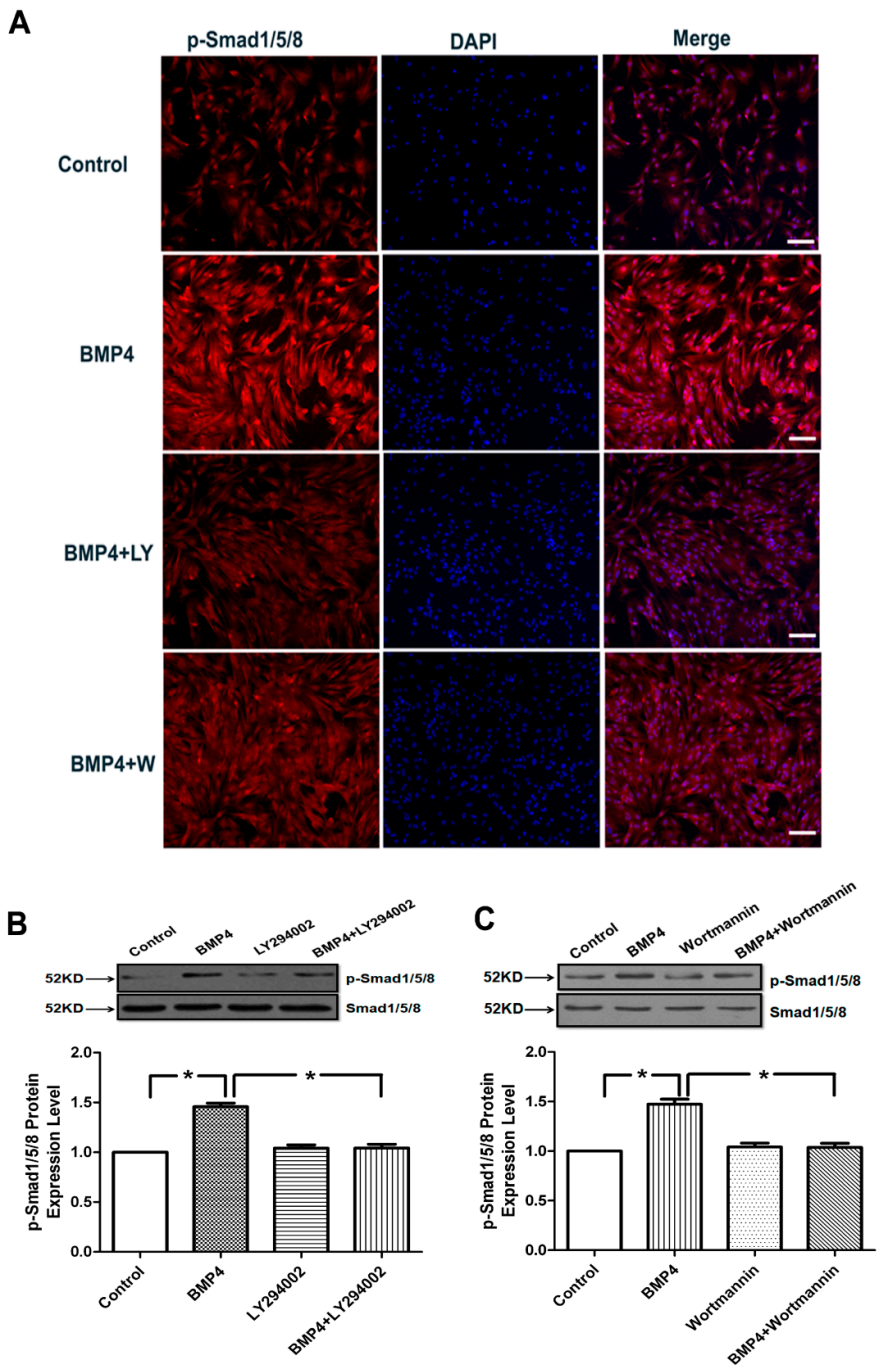
Effects of BMP4 on activation of Smad1/5/8. (**A**) Cells were fixed and stained with anti-p-Smad1/5/8 and the nucleus was staining with 4',6-diamidino-2-phenylindole (DAPI). The phosphorylation of Smad1/5/8 was activated by BMP4 treatment in rat PASMCs, but the effect was eliminated by PI3K/AKT inhibitors, LY294002 and wortmannin. Scale bar = 10 µm; (**B**,**C**) Incubation of PASMCs with BMP4 led to phosphorylation of Smad1/5/8. The phosphorylation of Smad1/5/8 activated by BMP4 was partly inhibited by LY294002 and wortmannin (PI3K/AKT inhibitors). All values are denoted as means ± SEM from three or more independent batches of cells. “LY” means LY294002; “W” means wortmannin. * *p* < 0.05.

BMP4 signal transduction is dependent on Smad1/5/8 phosphorylation. To determine the effect of BMP4 on Smad1/5/8 signaling, we performed immunofluorescence and Western blotting analysis on PASMCs. As shown by immunofluorescence and Western blotting (Figure 5A–C), the phosphorylation of Smad1/5/8 was activated by BMP4 treatment in rat PASMCs, but the effect was eliminated by PI3K/AKT inhibitors, LY294002 and wortmannin. The effect of BMP-4 may be BMPR-independent. Hence we examined the downstream effect of BMP-4 with knocking down of BMPR-2. The results showed that both BMP4 and BMP4 + si-BMPR-2 could activate the phosphorylation of Smad1/5/8, but when BMP4 + si-BMPR-2 was treated with PI3K/AKT inhibitors LY294002 and wortmannin, the effect disappeared (Figure S3). These results demonstrate that BMP4 stimulation of the PASMCs results in the activation of the PI3K/AKT signaling that stimulates the phosphorylation of transcription factors Smad1/5/8.

### 2.7. Discussion

Apoptosis is important in development, tissue homeostasis, and remodeling. Apoptosis also plays a fundamental role in the genesis of various diseases. It has been shown that pulmonary vascular medial hypertrophy is caused by imbalanced PASMCs proliferation and/or apoptosis [3,5,6]. However, the precise mechanisms participated in the regulation of PASMCs proliferation and apoptosis in PAH are still not completely clear. The present study provides a new piece of evidence that BMP4, activated and increased by hypoxia, inhibits the apoptosis of PASMCs through the PI3K/AKT pathway. BMP4-protected apoptosis of PASMCs is characterized by marked activation of AKT phosphorylation and enhanced p-Smad1/5/8 protein expression, by stimulation of an extrinsic cell death signaling pathway via down-regulation of effector caspases-3 expression and by mitochondria-dependent intrinsic pathway attenuating mitochondrial depolarization and up-regulation of the expression of Bcl-2.

A growing body of recent evidence suggests that BMP4 plays an important role in the pulmonary fibrosis and vascular remodeling process, including proliferation, cell migration, and apoptosis [8,12,13,14]. BMPR1A and BMPR2 are involved in the signaling transduction of BMP4. The binding of BMP4 to BMPR2 triggers the recruitment and phosphorylation of BMPR1A. BMPR1A subsequently causes downstream Smad-dependent and Smad-independent signaling transduction [16]. BMP4 is thought to play a key role in the pathogenesis of PAH. Although previous studies have demonstrated that the expression of BMPR2 decreases in lung tissue and distal small arteries in heritable PAH patients [13], there are many kinds of hypoxic pulmonary hypertension animal models, but so far no one has been able to imitate the pathophysiologic process found in humans. In our study, we used an animal model during a four-week period in a hypoxic environment and chose the peripheral arteries to examine the expression of BMPR2, as studies have shown that site-specific responses to BMPs are involved in PASMCs. BMP4 inhibited the proliferation of PASMCs isolated from proximal pulmonary arteries, but stimulated proliferation of PASMCs from peripheral arteries [9,13]. Although, both peripheral and proximal pulmonary arteries mediated the pulmonary vascular remodeling induced by hypoxia, the underlying cellular and molecular mechanisms appear to be different. The expression of BMPR2 depends on the species, sex, and the developmental stage at which the exposure to hypoxia occurred. In our study, we focus on the proliferation effect of PASMCs from peripheral arteries. Maybe there is an important reason that the expression of BMPR2 is not consistent with previous studies. In the present study, we found that BMP4 and BMPR2 mRNA and protein expression levels were significantly increased in hypoxia pulmonary arteries compared with controls. BMP4 protects rat PASMCs from apoptosis in a concentration-dependent manner.

It is well known that AKT is a serine/threonine protein kinase, which is activated by a number of growth factors and cytokines in a PI3K-dependent manner [28]. Activation of the PI3K/AKT pathway has a major impact on cell survival and apoptosis [20,29]. In mammalians, three isoforms of AKT have been proven: AKT1, AKT2, AKT3. The three isoforms have an 80% amino acid sequence homology [30,31]. It has been found that AKT1 is expressed in endothelial cells, which is the major isoform of endothelial cell AKT [32]. AKT2 is important to regulate heterotypic cell–cell interactions during vascular inflammation [33] AKT3 plays a pivotal role in atherosclerosis [34]. Therefore, the AKT signaling pathway regulates multiple cellular functions in cardiovascular disease. Here, we explored the role of AKT during PAH. In the present study, we have found that BMP4 induces phosphorylation of AKT and that the protective effect of BMP4 on cell viability is significantly blocked by PI3K/AKT inhibitors (LY294002 and wortmannin), indicating that the PI3K/AKT pathway is required for the anti-apoptotic effects of BMP4 in rat PASMCs. In addition, caspases are key mediators of programmed cell death (apoptosis). Among them, caspase-3, a frequently activated death protease, catalyzes the specific cleavage of many key cellular proteins [35]. In our studies, changes in the expression of procaspase-3 and cleaved caspase-3 indicate that BMP4, through the PI3K/AKT signal pathway, inhibits the SD-induced apoptosis by mitochondria-dependent ways in PASMCs.

BMP receptors are members of the TGF-β family receptors. BMP4 binds to its Type I and Type II receptors and subsequently phosphorylates Smad1 as well as Smad5 and Smad8 at their corresponding sites [36]. In this study, we found that BMP4 induced phosphorylation and hence activation of Smad1/5/8 with translocation of the activated Smad1/5/8 into the nucleus. Activation Smad1/5/8 promoted cell survival by PI3K/AKT signaling.

In previous studies, it has been shown that serum deprivation triggers apoptotic responses through mitochondrial pathways leading to mitochondrial dysfunction. Detection of mitochondrial permeability events provides early indication of the initiation of cellular apoptosis [37]. The inhibitory effect of BMP4 on the SD-induced loss of membrane potential is blocked in the presence of LY294002 or wortmannin. Geraci *et al*. [38] showed that the mRNA expression of Bcl-2 was up-regulated in lung tissues from sporadic and familial primary pulmonary hypertension (PPH) patients, Our results also show that BMP4 enhances the expression of Bcl-2 not only at mRNA level but also at the protein level.

## 3. Experimental Section

### 3.1. Materials

Recombinant human BMP4 was obtained from PeproTech (Rocky Hill, NJ, USA). Antibodies against procaspase-3, Bcl-2, caspase-3 were purchased from Santa Cruz Biotechnology Inc. (Santa Cruz, CA, USA). *BMPR2* siRNA (h) was obtained from Santa Cruz Biotechnology Inc. (Santa Cruz, CA, USA). Rabbit polyclonal antibodies to Smad1/5/8, phospho-Smad1/5/8, AKT, phosphor-AKT, BMP4, BMPR1A, BMPR2 were from Cell Signaling Technology, Inc. (Beverly, MA, USA). JC-1 probe and caspase-3 activity kit were obtained from Beyotime Institute of Biotechnology (Haimen, China). AKT1, phosphor-AKT1, AK2, phosphor-AKT2 were kindly provided by the department of pharmacology, Dalian Medical University. The Human BMP-4 ELISA (Enzyme-Linked Immunosorbent Assay) kit was purchased from Sigma-Aldrich. Enhanced chemiluminesence (ECL) reagents were from Amersham International (Amersham, UK). All other reagents were from common commercial sources.

### 3.2. Animals and Lung Tissues Preparation

Adult female/male Wistar rats with a mean weight of 200 g were from the Experimental Animal Center of Dalian Medical University (Grade II), Dalian, China. The animal protocols were approved by the Ethical Committee of Laboratory Animals at Dalian Medical University in January 2014. The animals were conditioned at a controlled ambient temperature of 22 ± 2 °C with 50% ± 10% relative humidity and at a 12-h light–dark cycle (lights on at 8:00 a.m.). Standard rat chow and water *ad libitum* were provided to all rats. Adult Wistar rats were randomized to four weeks of normal and hypoxic environments with fractional inspired oxygen (FiO_2_) 0.21 and 0.12, respectively, as previously described [39]. Normoxic rats were kept in the same room adjacent to the hypoxic chamber. At the end of the hypoxia exposure period, we anesthetized each rat with pentobarbital injection (120 mg/kg, i.p.), opened the thorax and removed the heart and lungs to the flat plate.

### 3.3. Cell Culture

The peripheral arteries were recovered from the lungs of adult rats. Segments of the PAs were cut open and mechanically stripped of adventitia and endothelium. PASMCs were dispersed according to our previously published protocol [39]. Cells were cultured in 20% fetal bovine serum (FBS)–Dulbecco’s modified eagle medium (DMEM) and in a 37 °C, 5% CO_2_ humidified incubator. The purity of PASMCs in the primary cultures was determined by specific monoclonal antibodies raised against smooth muscle β-actin (Boehringer Mannheim GmbH, Mannheim, Germany). Passages two to three were used for further experimentations. Before each experiment, the apoptosis in PASMCs was induced by serum deprivation, and the cells were incubated in DMEM without serum for 24 h. Then some cells were treated with the different experiment group. The HPASMCs and HPAECs were purchased from Lonza (Basel, Switzerland). HPASMCs and HPAECs were cultured according to the supplier’s instructions.

### 3.4. Real-Time Quantitative RT-PCR (qPCR)

RNAs were extracted from PASMCs using Trizol reagent and then determined by ultraviolet spectrophotometry (absorbance at 260 nm/280 nm; Invitrogen, Carlsbad, CA, USA). Total RNAs were reverse-transcribed using Superscript First-Strand Synthesis System for RT-PCR according to the manufacturer’s protocol. cDNA was reverse-transcribed from 0.5 μg of total RNA in a 10 μL reaction containing 5× PrimerScript^®^ Buffer 2 μL (Invitrogen), PrimerScript^®^ RT Enzyme Mix Ι 0.5 μL (Invitrogen), Oligo dT Primer (50 μΜ) × 10.5 μL (Invitrogen), Random six mers (100 μΜ) × 12 μL (Invitrogen). The cycling conditions were 95 °C for 5 min followed by 30 cycles of 45 s at 95 °C, 45 s at 52 °C, 75 s at 72 °C, and a final elongation for 10 min at 72 °C. qPCR was performed with an Applied Biosystems 7300 Fast Real-Time PCR system (Applied Biosystems, Foster City, CA, USA). Primers were specifically designed using Applied Biosystems Primer Express 3.0 (Applied Biosystems) and are listed in Table 1. The specificity of the primers was confirmed with a BLAST program (BLAST means the basic local alignment search tool which finds regions of local similarity between sequences. The program compares nucleotide or protein sequences to sequence databases and calculates the statistical significance of matches). Each 20 μL reaction contained 1× SYBR^®^ Premix Ex Taq™ II, 10 μM forward and reverse primers, 0.4 μL ROX reference dye, and 2 μL of cDNA. ABI 7300 Sequence Detector (Applied Biosystems) was programmed for the PCR conditions: 95 °C for 30 s, 40 cycles of 95 °C for 5 s, and 60 °C for 31 s, followed by routine melting curve analysis. Relative quantitation (RQ) of target gene expression was calculated by the 2^−ΔΔ*C*t^ method [40]. The first step in the RQ analysis is to normalize target gene expression level to β-actin (Δ*C*_t_). The second step is to compare the difference between normalized target gene expression in BMP4-treated and untreated samples (ΔΔ*C*_t_). Each experiment was repeated two to three times for three to four samples.

**Table 1:** Primer sequences used in real-time reverse transcription-polymerase chain reaction (RT-PCR).

Gene	Primer Sequences (5' to 3')	PCR Product Size	Accession Number
*Bcl-2*	Forward: 5'-CGGGAGAACAGGGTATGA-3'	149 bp	NM: 016993
	Reverse: 5'-CAGGCTGGAAGGAGAAGAT-3'		
*Caspas3*	Forward: 5'-CTACCGCACCCGGTTACTAT-3'	133 bp	NM: 012922.2
	Reverse: 5'-TTCCGGTTAACACGAGTGAG-3'		
*β-Actin*	Forward: 5'-AGGCCCCTCTGAACCCTAAG-3'	118 bp	EF: 156276.1
	Reverse: 5'-CCAGAGGCATACAGGGACAAC-3'		

### 3.5. Western Blot Analysis

The cells in six well culture plates were growth-arrested for 24 h before adding vehicle, BMP4 (100 ng/mL), LY294002 (10 μM), wortmannin (50 nmol) or BMP4 plus LY294002, BMP4 plus wortmannin in serum deprivation conditions. The cells cultured in complete medium were considered as control. After the treatment for 24 h, the cells were washed three times with ice-cold PBS, and then treated in 400 μL lysis buffer (Tris 50 mM, pH 7.4, NaCl 150 mM, TritonX-100 1%, EDTA 1 mM and PMSF 2 mM) and incubated for 30 min on ice. The lysates were sonicated for 1 min and then centrifuged at 14,000 r.p.m. for 15 min at 4 °C. The protein concentrations in the supernatant were confirmed using the Bio-Rad protein assay kit (Bio-Rad Laboratories, Inc., Berkeley, CA, USA). The protocol for Western blot was similar to previously described [39].

### 3.6. MTT Assay

The protocol of MTT assay was carried out according to the method published by Zhang *et al.* [39]. PASMCs were cultured in 96-well plates (about 1 × 10^4^ per well), and then the cells were subjected to growth arrest for 24 h before being placed in either complete medium (DMEM with 20% FBS) or switched to DMEM without serum for the next 24 h. The cells of each experimental group are treated with different drugs respectively. After 24 h of the incubation in 37 °C, the cells were incubated for 4 h in a medium containing 0.5% 3-(4,5-dimethylthiazol-2-yl)-2,5-diphenyl-tetrazolium bromide (MTT). Thereafter, the supernatant was removed, and dimethyl sulfoxide (150 μL/well) was added. The plates were then agitated on a plate shaker for 10 min at room temperature. The absorbance was read at 490 nm in a spectrophotometer.

### 3.7. Measurement of Caspase-3 Activity

Ac-DEVD–pNA (acetyl-Asp–Glu–Val–Asp p-nitroanilide), a caspase-3 substrate, was examined for caspase-3 activity, and the 405 nm absorbance was applied as directed by the kit. The protein samples were prepared as indicated in Western blot analysis. Then a reaction buffer containing Ac-DEVD–pNA (2 mM) were added to supernatants containing 50 μg of total protein, and the mixture was incubated for 4 h at 37 °C. The absorbance of yellow pNA was measured at 405 nm. The specific caspase-3 activity was normalized for total protein and then expressed as fold of the baseline caspase-3 activity of control cells cultured in DMEM with 10% FBS.

### 3.8. Mitochondrial Depolarization Assay

Mitochondrial function was indirectly assessed with the mitochondrial transmembrane potential measured with JC-1 red flpotential . Relative mitochondrial mass was measured by a fl Relative microscope (Nikon, Tokyo, Japan) using JC-1, analyzed for green fluorescence. Briefly, cells were cultured in six-well plates. After indicated treatments, they were incubated with an equal volume of a JC-1 staining solution (5 μg/mL) at 37 °C for 20 min and rinsed twice with PBS. Mitochondrial membrane potentials were monitored by determining the relative amounts of dual emissions from mitochondrial JC-1 monomers or aggregates using a flC for 20 microscope at 488 nm excitation. Mitochondrial depolarization was indicated by an increase in the green/red flat 488 nm intensity ratio.

### 3.9. Immunofluorescence

For immunofluorescence, PASMCs were grown on coverslips up to 80% confluence followed by serum starvation. Cells were fixed with 4% paraformaldehyde for 30 min and washed with cold PBS, then permeabilized in 0.01% Triton-X 100 for 5 min, and blocked with 5% normal bovine serum for 30 min. Cells were then incubated with p-Smad1/5/8 antibody at a dilution with 1:500 overnight at 4 °C. After PBS washing, cells were incubated with the secondary antibody IgG conjugated with rhodamine at a 1:500 dilution for 1 h at room temperature. DAPI (4,6-diamidino-2-phenylindole) was added to label nuclei, and the cells were then examined under a fluorescence microscope (Nikon).

### 3.10. Statistical Analysis

Results are shown as the mean ± SEM. One-way ANOVA and *t* test analysis (two-tailed) were used to determine the significance of differences between the means of different groups. A *p* value less than 0.05 was considered statistically significant.

## 4. Conclusions

In conclusion, our results have shown that BMP4 inhibits apoptosis of PASMCs via the PI3K/AKT pathway. This study establishes the signal transduction pathway and the mechanism of BMP4-inhibited PASMCs apoptosis. Furthermore, the regulation of the PI3K/AKT pathway by BMP4 may be an important mechanism underlying the treatment of pulmonary artery hypertension and provide a novel therapeutic insight for the future.

## Supplementary Files

Supplementary File 1
