# Supplementary material for: BMP4 Protects Rat Pulmonary Arterial Smooth Muscle Cells from Apoptosis by PI3K/AKT/Smad1/5/8 Signaling
Source: Int J Mol Sci. 2014 Aug 8;15(8):13738–54. doi: 10.3390/ijms150813738 (PMC4159822; doi:10.3390/ijms150813738)

Supplementary Information

Figure S1. (**A**–**C**) AKT isoforms protein expression in rat pulmonary arteries homogenates from normoxic and hypoxic rats; (**D**,**E**) BMP4 induced activation of AKT1, AKT2 in PASMCs. BMP4 up-regulated the expression of phosphorylation of AKT1 and AKT2 in a concentration-dependent manner. All values are denoted as the mean ± SEM from at least three separate experiments. * $p < 0.05$; ** $p < 0.01$.

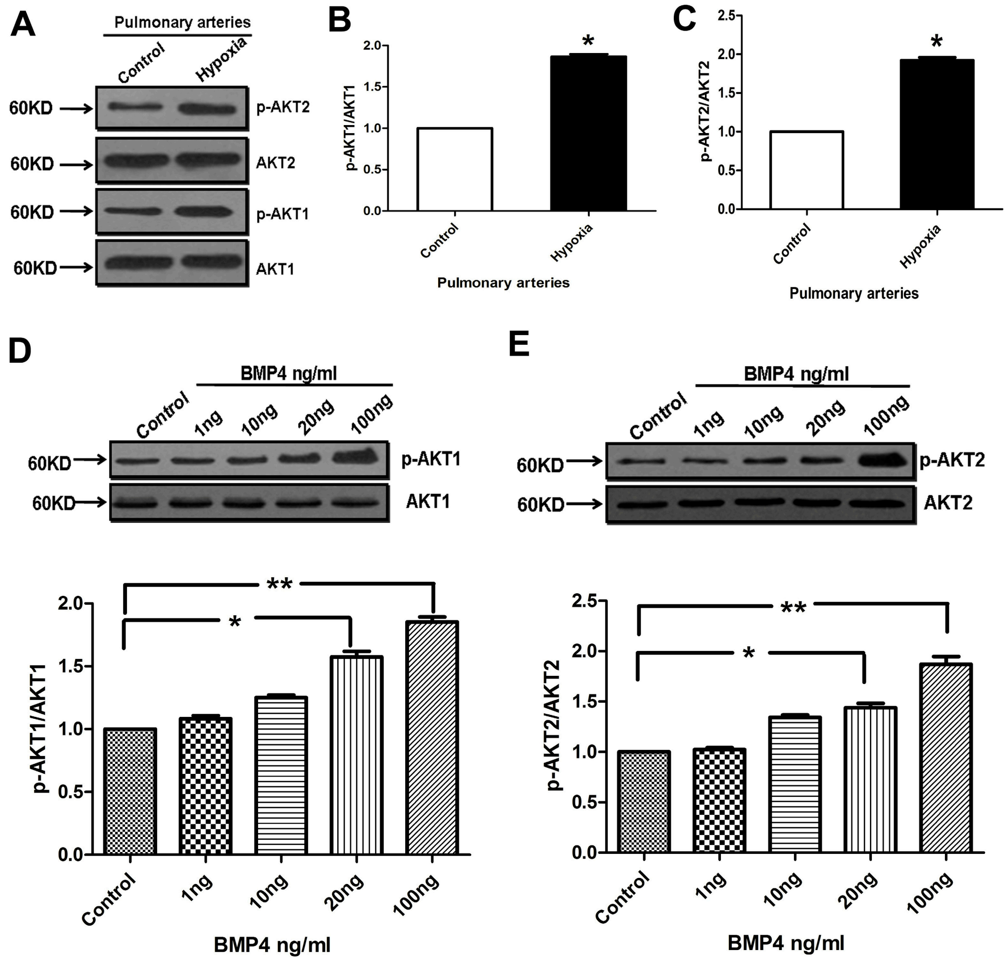

Figure S2. (**A**,**B**) The effect of hypoxia on BMP receptors expression in different time course. Cultured HPASMCs were exposed to hypoxia for 0 h as control, 12, 24, 48, 72 h respectively; hypoxia increased the protein expression of BMPR2 in a time-dependent manner. There were no significant differences in the protein levels of BMPR1A; (**C**) Hypoxia induced the generation of endogenous BMP4 in HPAECs in a time-dependent manner. All values are denoted as the mean ± SEM from at least three separate experiments. * $p < 0.05$; ** $p < 0.01$.

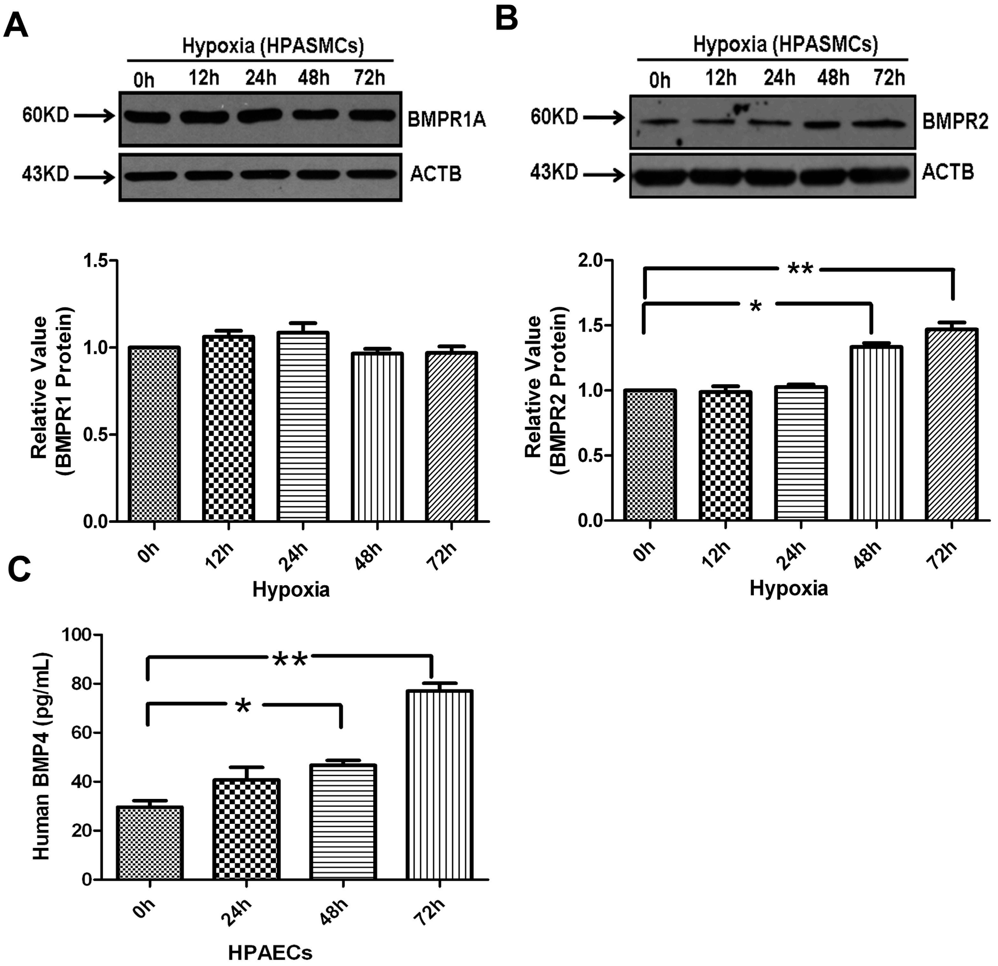

Figure S3. Effects of knockdown of the expression of *BMPR2* on the activation of Smad1/5/8. Cells were fixed and stained with anti-p-Smad1/5/8 and the nucleus was stained with DAPI. The phosphorylation of Smad1/5/8 was activated by BMP4 treatment in rat PASMCs. On the basis, we treated PASMCs with si-BMPR2, and we found that the activation of p-Smad1/5/8 still existed, but the effect was eliminated by the PI3K/AKT inhibitors LY294002 and wortmannin. Scale bar =10 μm.

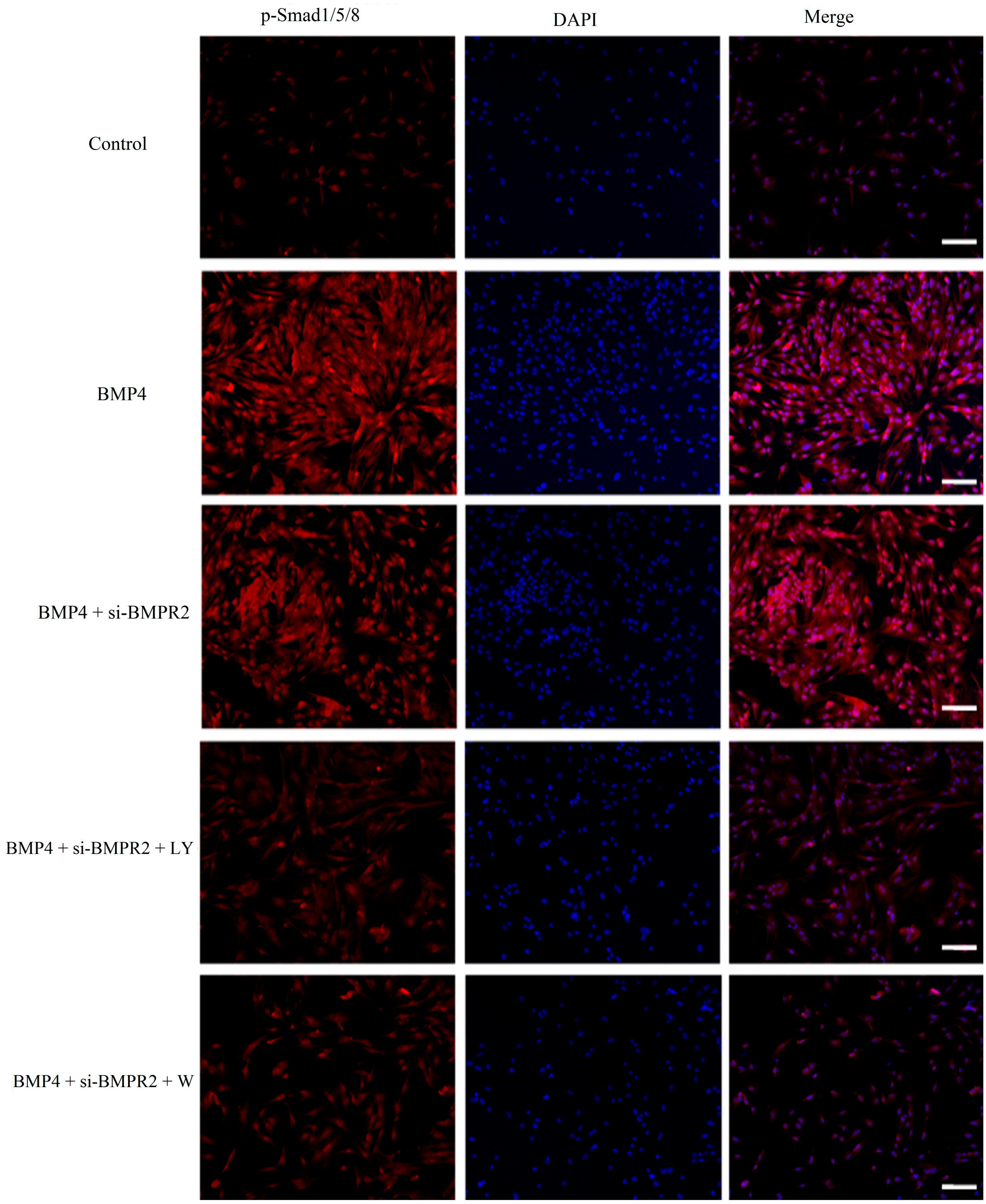

Supplement: Supplementary File 1 [file ijms-15-13738-s001.pdf]
